# Complete chloroplast genome sequence of *Solanum hjertingii*, one of the wild potato relatives

**DOI:** 10.1080/23802359.2022.2068983

**Published:** 2022-04-25

**Authors:** Tae-Ho Park

**Affiliations:** Department of Horticulture, Daegu University, Gyeongsan, South Korea

**Keywords:** Chloroplast, genome, genome sequence, *Solanum hjertingii*

## Abstract

*Solanum hjertingii* is a wild tuber-bearing species classified in the Solanaceae family. The chloroplast genome of *S. hjertingii* was completed via *de novo* assembly using Illumina paired-end sequencing data. Total length of the chloroplast genome of *S. hjertingii* is 155,545 bp consisting of 85,976 bp in a large single copy, 18,383 bp in a small single copy, and 25,593 bp in a pair of inverted repeat regions. Its structure is circular and typically quadripartite. It contains 158 predicted genes in total, including 105 protein-coding, 45 tRNA, and eight rRNA genes. Maximum likelihood phylogenetic analysis of the sequence along with 33 species in the *Solanaceae* family revealed that *S. hjertingii* belongs to a large clade with other *Solanum* species including *S. tuberosum* and is most closely grouped in the clade with *S. hougasii* and *S. stoloniferum* in the clade.

The wild tuber-bearing *Solanum hjertingii* Hawkes 1963 is a relative of the potato (*Solanum tuberosum*) originating from Mexico. It has been identified as a potential source of resistance to blackspot bruising because it exhibits neither enzymatic browning nor blackspot caused by impact or compression damage during harvest or storage (Hawkes 1990; Sim et al. 1997; Culley et al. 2002; Hara-Skrzypiec and Jakuczun 2013). It was also determined in contemporary researches to be resistant to both biotic and abiotic stresses such as *Phytophthora infestans*, drought and salt (data not shown). For these reasons, the wild species can be used for introgression of certain traits into the cultivated potatoes. However, *S. hjertingii* and *S. tuberosum* are not conventionally crossable, although both are tetraploids. The endosperm balance numbers (EBNs) for these species are 2 and 4, respectively (Hawkes 1990; Ortiz and Ehlenfeldt 1992; Cho et al. 1997). As a result, more advanced methods such as bridge crossing and somatic hybridization can be used for potato breeding as applied with other wild *Solanum* species (Hermsen 1966; Hermsen and Ramanna 1973; Binding et al. 1982; Iwanaga et al. 1991; Park et al. 2005; Luthra et al. 2019). The bridge crossing method was applied once (Culley et al. 2002), but somatic hybridization has not yet been tried with *S. hjertingii*. Therefore, we have been trying it and developing molecular markers to identify cytoplasm genome composition after obtaining hybrids via somatic hybridization.

The wild *S. hjertingii* species (PI186559) was obtained from the Highland Agriculture Research Institute, South Korea (37^^^68′05.4″N 128^^^73′09.1″E) and the specimen deposited at the National Agrobiodiversity Center, South Korea (http://genebank.rda.go.kr/, Hyun-Jin Park, rosa2125@korea.kr) as voucher number IT301488. Chloroplast genome sequencing was performed via the Phyzen bioinformatics pipeline (Kim et al. 2015). Total genomic DNA was isolated from one of the *S. hjertingii* lines by using a Genomic DNA Extraction kit for plants (RBC, New Taipei City, Taiwan). An Illumina paired-end (PE) genomic library was constructed with the genomic DNA by following the PE standard protocol (Illumina, San Diego, USA) and was sequenced at Macrogen (http://www.macrogen.com/kor/) using an Illumina HiSeq2000 platform. Approximately 1.48 Gbp of the sequence raw data obtained in total was trimmed and low-quality bases with a raw Phred score of 20 or less were removed using the CLC quality trim program in the CLC assembly cell package version 4.2.1 (CLC Inc, Rarhus, Denmark). Finally, approximately 1.27 Gbp of high-quality PE reads were applied for de novo assembly using the CLC de novo assembly program in the same package followed by retrieving the principal contigs representing the chloroplast genome and arranging the representative chloroplast contigs using Nucmer (Kurtz et al. 2004) and BLASTZ analysis (Schwartz et al. 2003) with the chloroplast genome sequence of *S. hougasii* (MF471372, Cho et al. 2018; Kim and Park 2020a). Gene annotation and manual curation were performed with the GeSeq program (Tillich et al. 2017) and BLAST searches. Phylogenetic analysis was performed using the chloroplast coding sequences of *S. hjertingii* and 33 published species belonging to the Solanaceae family by using a maximum likelihood method with the Kimura 2-parameter model and 1,000 bootstrap options in MEGA 6.0 (Tamura et al. 2013).

Total length of the complete *S. hjertingii* chloroplast genome (MK690623) is 155,545 bp consisting of a large single copy (LSC) region of 85,976 bp, a small single copy (SSC) region of 18,383 bp, and a pair of inverted repeat (IRa and IRb) regions of 25,593 bp with the typical circular and quadripartite structure like most plastids. Overall GC content was 37.88%. The closest *Solanum* species were *S. hougasii* (MF471372) and *S. stoloniferum* (MF471373, Park 2018; Kim and Park 2020b) each with a very high sequence identity of 99.97% and 99.96%, respectively. A total of 158 genes were annotated with an average length of 583.3 bp and gene features were typically identical to those of higher plants. The chloroplast genome consists of 105 protein-coding genes, 45 tRNA genes and eight rRNA genes with average sizes of 765.0 bp, 62.0 bp and 1131.0 bp, respectively.

Results from phylogenetic analysis revealed that *S. hjertingii* belongs to the same clade with other *Solanum* species as expected, and is most closely grouped in the clade with *S. hougasii* and *S. stoloniferum* (Figure 1). These results can be explained by the fact that the plastid DNA data generated a four-clade phylogeny and the three species originating from Mexico belong to the same clade (Spooner et al. 2008). Their genome compositions evolutionarily identified by gene and genomic in situ hybridization (GISH) analyses also support an AABB genome constitution (allotetraploid) for *S. hjertingii* and *S. stoloniferum*, and AABBPP (allohexaploid) for *S. hougasii* (Spooner et al. 2008; Pendinen et al. 2012; Ono et al. 2016).

**Figure 1.**
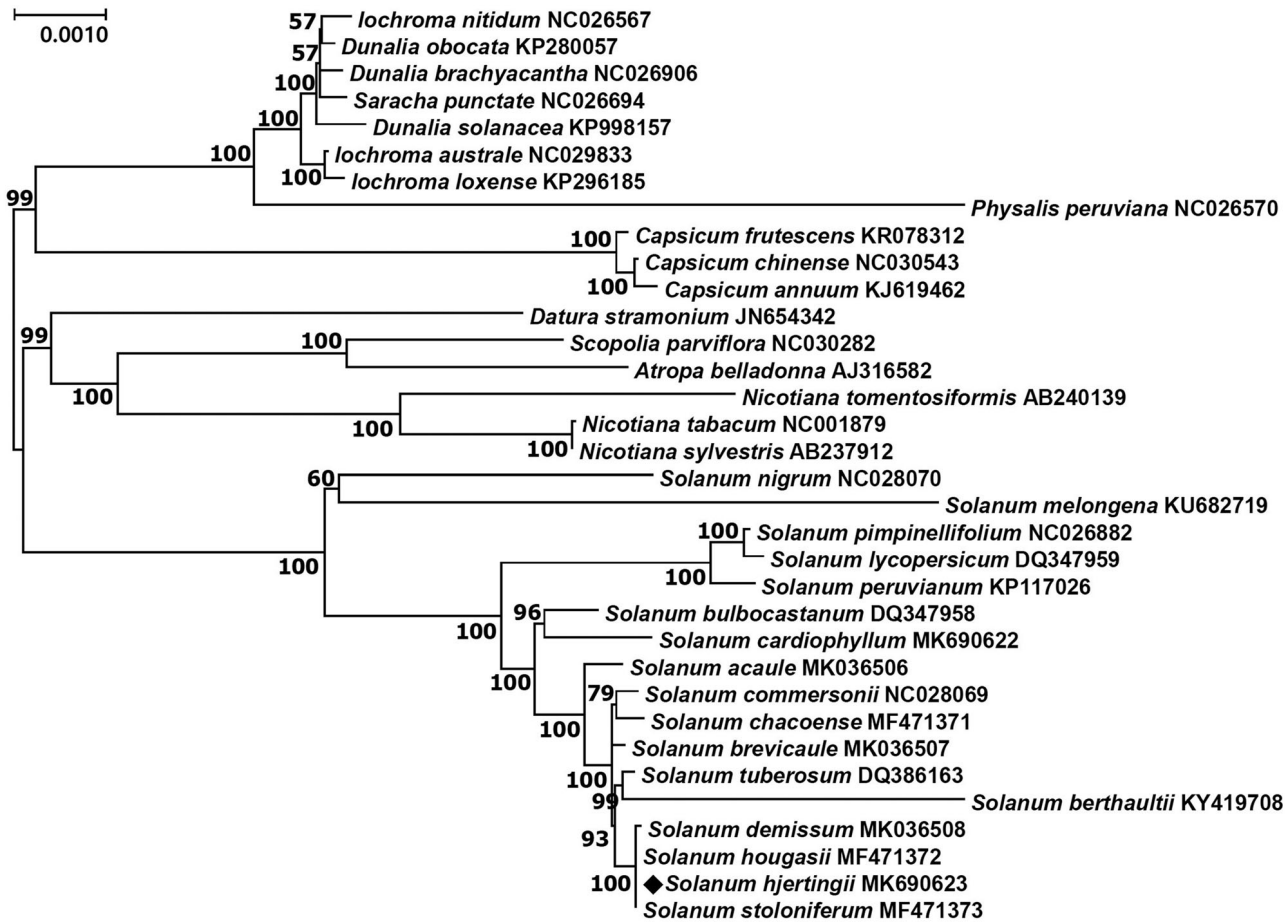
Maximum likelihood phylogenetic tree of *S. hjertingii* with 33 species belonging to the Solanaceae family based on chloroplast protein coding sequences. Numbers in the nodes are bootstrap values from 1000 replicates. The data were partially adopted from Park (2021).

## Data Availability

The data that support the findings of this study are openly available in the NCBI under accession number MK690623 (https://www.ncbi.nlm.nih.gov/nuccore/MK690623). The associated BioProject, SRA, and BioSample numbers are PRJNA704091 (https://www.ncbi.nlm.nih.gov/bioproject/PRJNA704091), SRR13766187 (https://www.ncbi.nlm.nih.gov/sra/SRR13766187), and SAMN18029792 (https://www.ncbi.nlm.nih.gov/biosample/SAMN18029792), respectively.

## References

[CIT0001] Binding H, Jain SM, Finger J, Mordhorst G, Nehls R, Gressel J. 1982. Somatic hybridization of an atrazine resistant biotype of *Solanum nigrum* with *Solanum tuberosum :* Part 1: Clonal variation in morphology and in atrazine sensitivity. Theor Appl Genet. 63(3):273–277. 24270827 10.1007/BF00304007

[CIT0002] Cho HM, Kim-Lee HY, Om YH, Kim JK. 1997. Influence of endosperm balance number (EBN) in interploidal and interspecific crosses between *Solanum tuberosum* dihaploids and wild species. Korean J Breed. 29:154–161.

[CIT0003] Cho K-S, Cho J-H, Im J-S, Choi J-G, Park Y-E, Jang D-C, Hong S-Y, Park T-H. 2018. The complete chloroplast genome sequence of *Solanum hougasii*, one of the potato wild relative species. Mitochondr DNA Part B. 2:755–757. 10.1080/23802359.2018.1491342 PMC7800044 33474312

[CIT0004] Culley DE, Dean BB, Brown CR. 2002. Introgression of the low browning trait from the wild Mexican species *Solanum Hjertingii* into cultivated potato (*S. tuberosum* L.). Euphytica. 125(3):293–303.

[CIT0005] Hara-Skrzypiec A, Jakuczun H. 2013. Diploid potato hybrids as source of resistance to blackspot bruising. Am J Potato Res. 90(5):451–459.

[CIT0006] Hawkes JG. 1990. The potato: Evolution, biodiversity and genetic resources. London: Belhaven Press.

[CIT0007] Hermsen JGT. 1966. Crossability, fertility and cytogenetic studies in *Solanum acaule* × *Solanum bulbocastanum*. Euphytica. 15(2):149–155.

[CIT0008] Hermsen JGT, Ramanna MS. 1973. Double-bridge hybrids of *Solanum bulbocastanum* and cultivars of *Solanum tuberosum*. Euphytica. 22(3):457–166.

[CIT0009] Iwanaga M, Freyre R, Watanabe K. 1991. Breaking the crossability barriers between disomic tetraploid *Solanum acaule* and tetrasomic tetraploid *S. tuberosum*. Euphytica. 52(3):183–191.

[CIT0010] Kim K, Lee SC, Lee J, Yu Y, Yang K, Choi BS, Koh HJ, Waminal NE, Choi HI, Kim NH, et al. 2015. Complete chloroplast and ribosomal sequences for 30 accessions elucidate evolution of *Oryza* AA genome species. Sci Rep. 5:15655. 26506948 10.1038/srep15655 PMC4623524

[CIT0011] Kim S, Park T-H. 2020a. Development of *Solanum hougasii*-specific markers using the complete chloroplast genome sequences of *Solanum* species. J Plant Biotechnol. 47(2):141–149.

[CIT0012] Kim S, Park T-H. 2020b. Comparison of the complete chloroplast genome sequence of *Solanum stoloniferum* with other *Solanum* species generate PCR-based markers specific to for *Solanum stoloniferum*. J Plant Biotechnol. 47(2):131–140.

[CIT0013] Kurtz S, Phillippy A, Delcher AL, Smoot M, Shumway M, Antonescu C, Salzberg SL. 2004. Versatile and open software for comparing large genomes. Genome Biol. 5(2):R12. 14759262 10.1186/gb-2004-5-2-r12 PMC395750

[CIT0014] Luthra SK, Tiwari JK, Kumar V, Lal M. 2019. Evaluation of interspecific somatic hybrids of potato (*Solanum tuberosum*) and wild *S. cardiophyllum* for adaptability, tuber dry matter, keeping quality and late blight resistance. Agric Res. 8(2):158–164.

[CIT0015] Ono S, Sanetomo R, Hosaka K. 2016. Genetic transmission of *Solanum demissum* (2n = 6x = 72) chromosomes from a pentaploid hybrid of *S. tuberosum* (2n = 4x = 48) into aneuploid BC_1_ progeny. Euphytica. 207(1):149–168.

[CIT0016] Ortiz R, Ehlenfeldt MK. 1992. The importance of endorsperm balance number in potato breeding and the evolution of tuber-bearing *Solanum* species. Euphytica. 60(2):105–113.

[CIT0017] Park T-H. 2018. Chloroplast genome sequence of the wild tetraploid potato relative *Solanum stoloniferum*. Mitochondrial DNA Part B. 3(1):416–418. 33474189 10.1080/23802359.2018.1456983 PMC7799944

[CIT0018] Park T-H. 2021. Complete chloroplast genome sequence of the wild diploid potato relative *Solanum acaule*. Mitochondr DNA Part B. 3:1189–1191. 10.1080/23802359.2021.1902414 PMC8008929 33829083

[CIT0019] Park T-H, Vleeshouwers VG, Hutten RC, van Eck HJ, van der Vossen E, Jacobsen E, Visser RG. 2005. High-resolution mapping and analysis of the resistance locus *Rpi-abpt* against *Phytophthora infestans* in potato. Mol Breeding. 16(1):33–43.

[CIT0020] Pendinen G, Spooner DM, Jiang J, Gavrilenko T. 2012. Genomic *in situ* hybridization reveals both auto- and allopolyploid origins of different North and Central American hexaploid potato (*Solanum* sect. *Petota*) species. Genome. 55(6):407–415. 22594521 10.1139/g2012-027

[CIT0021] Schwartz S, Kent WJ, Smit A, Zhang Z, Baertsch R, Hardison RC, Haussler D, Miller W. 2003. Human–mouse alignments with BLASTZ. Genome Res. 13(1):103–107. 12529312 10.1101/gr.809403 PMC430961

[CIT0022] Sim SK, Ohmann SM, Tong CBS. 1997. Comparison of polyphenol oxidase in tubers of *Solanum tuberosum* and the non-browning tubers of *S. hjertingii*. Am Potato J. 74(1):1–13.

[CIT0023] Spooner DM, Rodríguez F, Polgár Z, Ballard HE, Jr, Jansky SH. 2008. Genomic origins of potato polyploids: GBSSI gene sequencing data. Crop Sci. 48:S27–S36.

[CIT0024] Tamura K, STecher G, Peterson D, Filipski A, Kumar S. 2013. MEGA6: molecular evolutionary Genetics Analysis version 6.0. Mol Biol Evol. 30(12):2725–2729. 24132122 10.1093/molbev/mst197 PMC3840312

[CIT0025] Tillich M, Lehwark P, Pellizzer T, Ulbricht-Jones ES, Fischer A, Bock R, Greiner S. 2017. GeSeq – versatile and accurate annotation of organelle genomes. Nucleic Acids Res. 45(W1):W6–W11. 28486635 10.1093/nar/gkx391 PMC5570176

